# The antioxidative influence of dietary creatine monohydrate and L-carnitine on laying performance, egg quality, ileal microbiota, blood biochemistry, and redox status of stressed laying quails

**DOI:** 10.1016/j.psj.2023.103166

**Published:** 2023-10-10

**Authors:** Karrar Imad Abdulsahib Al-Shammari, Sarah Jasim Zamil, Justyna Batkowska

**Affiliations:** ⁎ Department of Animal Production Techniques, Al-Musaib Technical College, Al-Furat Al-Awsat Technical University, Babylon, Iraq; † Institute of Biological Bases of Animal Production, University of Life Sciences in Lublin, 20-950 Lublin, Poland

**Keywords:** antioxidant, egg, oxidative stress, physiology, poultry

## Abstract

The experiment was implemented to assess the influence of dietary supplementation of laying quails with creatine monohydrate (**CrM**), L-carnitine (**CAR**) and their mixture (**CrMCAR**) as antioxidants against oxidative stress (**OS**) induced by 2.5 ppm lead acetate (**LA**) in drinking water on productive, physiological and microbial aspects. In total, 400 laying quail females at 10 wk of age were divided into a randomized design with 5 groups and 4 replicates of 20 birds each. Birds were fed *ad libitum* with a balanced diet for 8 wk. The control group was kept under no-stress conditions and was given fresh water without any additives (G1). While birds in other groups were exposed to OS induced experimentally by 2.5 ppm LA in drinking water with no feed additive (G2) or supplemented with 500 mg/kg CrM (G3) or 500 mg/kg CAR (G4) or combination of 250 mg/kg each of CrM and CAR (CrMCAR, G5) to feed mixture.

Compared to G2, G5 demonstrated the reduction (*P* ≤ 0.05) of feed conversion ratio, feed intake, mortality and ileal total coliform, as well as serum and egg malondialdehyde and serum lipid hydroperoxide, uric acid, glucose, cholesterol, enzymatic activities (alanine aminotransferase, aspartate transaminase, alkaline phosphatase, creatine phosphokinase, γ-glutamyl transferase), and heterophils/lymphocytes ratio. In the meanwhile, there was an increase (*P* ≤ 0.05) in egg production, egg mass, and weight with the improvement of egg quality, serum sex hormones level and ileal lactic acid bacteria for G5 followed by G4 and G3. Moreover, G5 enhanced (*P* ≤ 0.05), the total antioxidant capacity of egg and serum glutathione, superoxide dismutase, catalase, glutathione peroxidase, protein and calcium levels. Therefore, dietary CrMCAR, CAR and CrM have analogous influence to control by improving the antioxidant and physiological parameters which resulted in better productive performance and egg characteristics of stressed quails. These antioxidants, especially in their equal combination, are beneficial to alleviate oxidative stress incidence and can be recommended for poultry feeding under various aspects of environmental stresses.

## INTRODUCTION

Many external stressors in avian species play a unique role in the passive modification of metabolic and physiological phenomena which is due to the overproduction the reactive oxygen and nitrogen species and their harmful free radicals *in vivo* (Estévez, 2015; Al-Shammari et al., 2019a). Oxidative stress (**OS**) gives a basic indicator during exposure to climatic, nutritional, chemical, physical, behavioral, and psychological stressors. The OS causes prolonged oxidative damage to essential parts of cells and living tissues through the endogenous imbalance of antioxidant and prooxidants pathways (Al-Shammari and Batkowska, 2021). Thus, a cooperatively complex network of internally and externally powerful antioxidants preserves the efficient redox system in the body and this could impact positively for activation of gene expression, homeostasis maintenance, regulation of cell signaling and nuclear transcription factors (Surai et al., 2019; Surai and Earle-Payne, 2022). The OS is characterized by its detrimental influence on various physiological characteristics of domestic poultry by decreased antioxidant profile and increased oxidative injury for cellular lipids, proteins, carbohydrates and genetic material (Al-Shammari et al., 2019a; Surai et al., 2019). To reduce the OS incidence and optimize antioxidant defense, many researchers attempted to find out various antioxidant materials incorporated in diet either from natural or artificial sources as optimal procedures to meet and maintain the physiological needs of poultry under stressful conditions.

Creatine is a nitrogenous constituent produced by cooperative metabolism in hepatic, renal and pancreatic tissues, which is composed of methionine, arginine, and glycine or from its precursor, guanidine acetic acid (**GAA**) (Wu, 2020). Phosphocreatine is a natural compound of creatine in muscle which exerts a pivotal importance during anaerobic stress as the source of energy essential for muscle fibers contraction, maintaining adenosine triphosphate (**ATP**) levels and reducing accumulation of lactic acid in muscle (Wyss and Kaddurah-Daouk, 2000; Zhang et al., 2014). Generally, in animal cells, adenosine diphosphate (**ADP**) accepts phosphate groups from phosphocreatine and, therefore, ADP is converted into ATP which has in turn ergogenic effects (Wang et al., 2015; Ibrahim et al., 2019). It was found that creatine or its derivative form, creatine monohydrate (**CrM**) used as a powerful feed additive by exhibiting its palliative and antioxidant properties against transport stress (Zhang et al., 2014, 2017) and thermal stress (Al-Tamimi et al., 2019) in broiler chickens, and more recently, to counteract the overall OS in Japanese quails (Al-Shammari, 2023).

L-carnitine (**CAR**) is a water-soluble product and free radicals scavenger with a multifunctional antioxidative mechanism where found in microorganisms and animal and plant cells. In animals, CAR is endogenously biosynthesized in the liver from precursor amino acids (methionine and lysine) with the existence of many vitamins as cofactors such as vitamins (C, B3, B6, and B9) and iron (Arslan, 2006; Surai, 2015). Higher demand for CAR from exogenous sources is increasingly required during enhanced metabolic efficiency and stressful environment to modify lipid and glucose metabolism in poultry (Arslan, 2006; Rehman et al., 2017). CAR is an indispensable molecule, biochemically linked with the transfer of long-chain fatty acids to oxidize them for energy generation in the mitochondrial matrix through the functional property of acyltransferases (Adabi et al., 2011). Also, CAR plays role in energy generation from glucose because of the regulatory influence of CAR on the mechanism of pyruvate dehydrogenase that controls energy pathways connected with the citric acid cycle, and glycolysis (Ringseis et al., 2018). It was well reported that dietary supplementation of CAR could promote growth performance and improve the hemato-biochemical status, redox reaction, immune system as well as the semen characteristics for broilers and layers under different specified doses and feeding durations (Adabi et al., 2011; Rehman et al., 2017; Çetin and Güçlü, 2020).

Based on our previously published data (Al-Shammari, 2023), we tested the single effect of CrM and CAR or their synergistic effect in the diet of meat-type Japanese quails under oxidative stress conditions. However, the same doses of these additives and the potential impacts as antioxidants have not been hypothesized on laying performance under similar conditions. Therefore, in the current experiment, several variables of the influences of dietary supplementation of CrM, CAR and their combination (CrMCAR) on productivity, eggs quality, ileal microflora, antioxidative status, and physiology of laying type Japanese quails exposed to induced oxidative stress were investigated.

## MATERIALS AND METHODS

This research was performed at the poultry house and labs of the Al-Musaib Technical College following the recommendations on the ethics of working on animals of the Scientific Committee in the Department of Animal Production Techniques, Al-Musaib Technical College, Al-Furat Al-Awsat Technical University, Babylon, Iraq.

In total, 400 healthy Japanese quail females at 10 wk of age with similar body weights (171 ± 0.4 g) were chosen and reared for 8 wk. Birds were randomly assigned into 5 groups and 4 replicates with 20 birds in each. The birds in each replicate were individually identified with wing marks and kept in 90 × 80 × 30 cm wire cages equipped with feeders and drinkers. The environmental conditions were automatically controlled with the provision of a comfortable temperature (24 °C), optimal ventilation, and humidity and with lighting program of 16 h of light:8 h of darkness/d. Birds were fed *ad libitum* with a balanced diet prepared in accordance with NRC (1994) to meet their nutritional requirements (Table 1).

**Table 1:** Diet composition and its chemical analysis.

Ingredients (%)		Chemical analysis	
Yellow corn	53.76	Crude protein (%)	19.15
Soybean meal	29.27	Metabolizable energy (kcal/kg)	2840
Sunflower oil	4.850	Calcium (%)	3.140
Table salt	0.310	Available phosphorus (%)	0.430
Limestone	9.500	Lysine (%)	1.060
Dicalcium phosphate	1.760	Methionine (%)	0.430
DL-methionine	0.200	Methionine+cysteine (%)	0.711
Premix^1^	0.350		
Total	100		

1 Vitamin-mineral premix (Provimi, Jordan) provides per 1 kg the following items: 5.9% crude protein, 3,600 kcal metabolizable energy, 9,000 IU vitamin E, 8,000 IU vitamin A, 3,000 IU vitamin D3, 3 g vitamin K, 0.02 g vitamin B12, 50 g niacin, 0.1 g biotin, 3 g thiamin, 1 g folacin, 15 g pantothenic acid, 10 mg riboflavin, 4 g pyridoxine, 1.5 mg menadione, 60 g zinc (zinc sulfate), 60 g manganese (manganous oxide), 10 g copper (copper sulfate), 50 g iron (iron sulfate), 0.42 g selenium (sodium selenite), 1 g iodine (ethylenediamine dihydroiodide), 0.5 g sodium molybdate, 20 g antioxidant.

Birds in the control group were kept under no-stress conditions and were given fresh drinking water without any additives (G1). While birds in other groups were exposed to OS induced experimentally by 2.5 ppm of lead acetate (**LA**) in drinking water with no feed additive (G2) or supplemented with 500 mg/kg CrM (G3) or 500 mg/kg CAR (G4) or combination of 250 mg/kg each of CrM and CAR (CrMCAR, G5) to feed mixture. LA (99% purity) was obtained from a commercial producer (Lab Tech Chemicals, Ginsheim-Gustavsburg, Germany) in powdery form (Al-Shammari and Batkowska, 2021; Al-Shammari, 2023). It was dissolved and prepared into pure drinking water and served to birds daily. The creatine monohydrate (**CrM**) and L-carnitine (**CAR**) (Shandong Longchang Animal Health Product Co., Jinan, China) were added singly or in combination to the diet daily. These dietary additives were in powdery form, mixed into diet thoroughly and homogeneously and then packaged in sealed polypropylene woven sacks to keep their antioxidant activity from probable noxious impacts of the external environment.

The productive traits such as feed intake (**FI**), water intake (**WI**), and mortality (**MORT**) were registered daily and their values are presented periodically every 4 wk (10–14 and 15–18 wk of birds age) for each replication in group. Eggs were collected, counted, and weighed daily in each cage and by multiplying egg weight (**EW**) with egg production, the egg mass (**EM**) per hen was calculated. The feed conversion ratio (**FCR**) was calculated by dividing the grams of FI by grams of egg mass or number of eggs. The average percentage values of hen-day egg production (**HD**) and HD as egg per hen in particular experimental periods were calculated (Al-Shammari et al., 2019b). The protein efficiency ratio (**PER**) was estimated as EM per crude protein intake, and the energy efficiency ratio (**EER**) was estimated as EM per 100 kcal of metabolizable energy intake. Moreover, average body weight (**BW**) was individually measured for each bird in replicate at 14th at 18th wk.

Internal and external egg quality were measured twice, after 4 and 8 wk of experiment by taking 40 fresh eggs from each group (*n* = 10/replicate). The following quality traits of egg were evaluated:

1. Whole egg
  • EW using a digital scale with an accuracy of 0.01 g;
  • egg shape index (**EI**) as a ratio of the equatorial axis to the longitudinal one using a digital vernier caliper;
  • the egg surface area (**ESA**) was calculated based on the formula (Duman et al., 2016)
    $$\text{ESA}\;\left(cm^{2}\right)=3.9782\;\times \text{EW}\;{\left(g\right)}^{0.7056}$$
2. Eggshell
  • eggshell weight (**SW**) using a digital scale with an accuracy of 0.01 g;
  • eggshell weight per unit of surface area (**SWUSA**) was estimated using the formula (Stanquevis et al., 2021)
    $$\text{SWUSA}=\left(\frac{\text{SW}\;\left(g\right)}{3.9782}\times \text{EW}\;\left(g\right)\right)\times \;100$$
  • eggshell thickness (**ST**) using a digital vernier caliper;
  • eggshell volume (**SV**) was obtained by multiplying the ESA (cm^2^) with ST (cm) as it was mentioned by Shafey (2002).
3. Albumen
  • albumen weight (**AW**) using a digital scale with an accuracy of 0.01 g;
  • albumen height (**AH**) using a digital vernier caliper;
  • Haugh unit (**HU**) was calculated based on Eisen et al. (1962);
4. Yolk
  • yolk weight (**YW**) using a digital scale with an accuracy of 0.01 g;
  • yolk index (**YI**) as a ratio of the yolk height to its diameter;
  • yolk color (**YC**) using the 16-point scale (DSM YolkFan, Basel, Switzerland).

Samples of yolk and albumen were homogenized separately well using a blender and cooled to 4 °C in 50 mM phosphate buffer and then centrifuged (Muchacka et al., 2018). The collected supernatants were stored at −20 °C and used for redox markers determination. Lipid peroxidation in the egg was estimated by measuring the level of malondialdehyde (**MDA**) in both egg albumen and yolk using thiobarbituric acid following the method of Draper and Hadeley (1990). The ferric-reducing ability of plasma (**FRAP**) assay was used for the determination of total antioxidant activity (**TAC**) expressed as mg gallic acid equivalents (**GAE**) per g of aqueous solutions of egg yolk and albumen based on a slightly modified procedure of Benzie and Strain (1996).

At the end of the experiment 4 birds from each replication were randomly selected and, after 10 h of starvation, blood samples were collected twice from the same bird. The first sample was taken by puncture of a brachial vein, the second one was collected immediately after slaughter from the jugular vein. One part of harvested blood was put in anticoagulant tubes containing K3EDTA to enumerate the heterophil to lymphocyte ratio (**H/L**) (Burton and Guion, 1968) and to investigate FRAP value in plasma, following the spectrophotometric methodology (Benzie and Strain, 1996). The other part of the collected blood was placed into a gel separator tube to separate serum by centrifugation at 3,000 RPM for 15 min. and thereafter all serum samples were preserved at −25°C until the execution time of other analyses.

The serum redox indicators involving MDA and lipid hydroperoxide (**LOOH**) were estimated according to Salih et al. (1987) and Södergren et al. (1998), respectively. The analytical procedures of Misra and Fridovich (1972) and Aebi (1984) were followed to determine the antioxidative enzyme sets such as superoxide dismutase (**SOD**), catalase (**CAT**), and glutathione peroxidase (**GPx**). The commercial kits (Sigma Aldrich, St. Louis, MO) were used to implement all redox analyses using a spectrophotometer (Shenzhen, China). Also, the quantitative colorimetric method based on guidelines of diagnostic bioassay kit (Bioxytech GSH-420, OXIS Research, Portland, OR) was used to measure glutathione (**GSH**) level.

Moreover, a spectrophotometric method was carried out by using respective commercial kits (Biolabo, Maizy, France) to determine the serum biochemical indices as follow: total protein (**TP**), calcium (**Ca**), phosphorus (**P**) (Young, 2000), creatinine (**CREA**), uric acid (**UA**), total cholesterol (**CHOL**), and alkaline phosphatase (**ALP**) (Burtis and Ashwood, 1999). A commercial kit (Cromatest, Barcelona, Spain) was also used for glucose value detection (Young, 2000). The standard protocol of Reitman and Frankel (1957) stated in the Randox enzymatic test kit (VirtualExpo Group, Marseille, France) was implemented to determine the activity of alanine aminotransferase (**ALT**) and aspartate transaminase (**AST**). Concerning the calculation of the activity of creatine phosphokinase (**CPK**) and gamma-glutamyl transferase (**GGT**), the spectrophotometric method proposed by Tietz (1986) was followed by a commercial kit (Ortho-Clinical Diagnostics, Raritan, NJ).

For hormonal analyses in serum, the enzyme-linked immunosorbent assay (**ELISA**) microplate reader (BK-EL10C, Biobase, Shandong, China) was run to register the absorbance according to the strict steps stated in commercially specific enzyme-radioimmunoassay kit (IDS, Boldon, UK) to quantify of corticosterone (**COR**) level. Also, respective kits of ELISA (Baolai Biotechnology Co., Ltd., Shandong, China) were used to determine sex hormones including estradiol (**E2**), progesterone (**P4**), follicle-stimulating hormone (**FSH**), luteinizing hormone (**LH**) following the methodology reported by the manufacturer.

In total, 40 birds were slaughtered according to euthanasia protocol. After slaughtering and dissection of the gut, 40 samples of ileum content (*n* = 2/replicate) were gently collected. Ileum contents were placed into sterile tubes and immediately used to examine the microbial population. After all microbial samples were subjected to anaerobic diluents of serial dilution (10^−4^–10^−6^) and inoculated with sterile agar, the procedure of Bryant and Burkey (1953) was followed to perform microbial analysis. For each selective culture, a specific agar was used to grow and account for the total aerobic bacteria and specific colony type of coliforms, lactic acid bacteria, *Escherichia coli, Salmonella* spp. (Manafi et al., 2016) and total fungal counts (Speak, 1984) under various times and temperatures of microbial plate incubation and inoculation. After enumeration, the microbial data per 1 g of ileum were converted and expressed as log10 colony-forming units (log10 CFU/g of wet weight).

Also, the digest (*n* = 2/replicate) were collected gently from gizzard, small intestine and large intestine segments separately and preserved at −20°C in Eppendorf-type tubes until pH determination. Briefly, by taking 1 g of content then mixing and shaking it with 2 g of distilled water and afterward recording 3 readings per content using a pH meter (PHS-550, Lohand Biological Co., Zhejiang, China) according to the procedure of Chaveerach et al. (2004).

The statistical analysis of the data obtained was performed SAS software (SAS, 2012). The differences among the experimental groups were verified using a general linear model and 1-way ANOVA with Duncan's multiple range test (Duncan, 1955) to compare the particular means. The significance level of *P* < 0.05 was applied using the following model: *Y_ij_ =μ +T_i_ +e_ij_*, where *Y_ij_* is the observation value; *μ* is the overall mean; *T_i_* is the treatment group effect (G1–G5), and *e_ij_* is the random error.

## RESULTS

In Table 2 the traits characterizing Japanese quails' productivity were shown. The best effect on the body weight at the end of particular experimental periods was achieved in birds from group G1 but also G5, treated with a combination of CAR and CrM against induced oxidative stress. Significantly the lowest feed intake (*P* ≤ 0.05) was found in the G2 group (positive control) with the highest in the case of FI in G1 (kept without any additives) and G3 (with CrM additive). Very similar relationships between groups were demonstrated by the water intake, the highest was estimated for G1 and G3 followed by G4, G5, and G2 exclusively influenced by oxidative stress. The FCR both calculated per g of egg as well as per egg, had the lowest values in group G5, given the combination of CAR and CrM against oxidative stress. The lowest PER characterized G2 with the highest in the G5 group irrespective of the analyzing period. Similar relationships between groups were stated in energy efficiency. The biggest EM (*P* ≤ 0.05) was produced by birds in the control group (G1), regardless of the experimental period, followed by groups with antioxidant factors (G5, G3, and G4) with the smallest in the OS group (G2). The highest egg production (HD) was achieved by quails in the negative control group (G1) and then in G5 (CrMCAR), G3 (CrM), G4 (CAR), and G2. In between 15 and 18 wk of age birds in all groups had lower HD values than in the first period (10–14 wk) probably resulting from the fact that birds were after the peak production. The best liveability (lowest MORT) was stated in G1 with the worst in the G2 group, all groups supplemented with antioxidant factors did not differ according to the substance used.

**Table 2:** The production parameters of Japanese quails fed with antioxidant factors against the oxidative stress.

Trait	Age (wk)	Group					SEM	*P* value
		G1	G2	G3	G4	G5		
BW (g)	14	179.75^a^	164.65^b^	166.75^b^	167.86^ab^	170.14^a^	12.65	0.032
	18	181.87^a^	166.80^c^	169.75^bc^	168.00^bc^	175.76^ab^	23.72	0.047
FI (g)	10–14	777.00^a^	665.28^b^	752.36^a^	755.44^a^	693.28^ab^	13.10	0.021
	15–18	830.20^a^	724.12^c^	807.12^a^	797.96^ab^	743.36^bc^	26.98	0.040
	Total	1607.2^a^	1389.4^c^	1559.4^a^	1553.4^ab^	1436.6^bc^	22.26	0.016
WI (mL)	10–14	2486.4^a^	2128.8^c^	2407.5^a^	2417.4^a^	2218.4^ab^	69.91	0.022
	15–18	3403.8^a^	2968.8^c^	3309.1^a^	3136.3^ab^	3047.7^bc^	43.76	0.031
	Total	5890.2^a^	5097.7^c^	5716.7^a^	5553.7^ab^	5266.2^b^	35.87	0.050
FCR (g feed/g egg)	10–14	2.43^ab^	3.19^a^	2.93^a^	3.07^a^	2.40^b^	0.210	0.034
	15–18	2.78^b^	3.66^a^	3.08^ab^	3.25^a^	2.68^b^	0.680	0.042
	Total	2.59^b^	3.35^a^	2.92^ab^	3.07^a^	2.46^b^	0.477	0.017
FCR (g feed/egg)	10–14	28.06^b^	31.88^a^	31.21^a^	31.76^a^	26.72^b^	2.651	0.023
	15–18	32.38^b^	35.60^a^	33.15^ab^	34.91^a^	30.97^b^	6.980	0.043
	Total	30.22^ab^	33.74^a^	32.18^a^	33.34^a^	28.85^b^	9.782	0.052
PER (g egg/g protein)	10–14	2.15^a^	1.63^b^	1.78^ab^	1.69^b^	2.18^a^	0.661	0.026
	15–18	1.88^a^	1.43^b^	1.69^ab^	1.61^ab^	1.95^a^	0.090	0.045
	Total	2.01^a^	1.53^b^	1.79^a^	1.70^ab^	2.12^a^	1.001	0.016
EER (g egg/100 kcal)	10–14	14.48^a^	11.02^b^	12.02^ab^	11.46^b^	14.67^a^	8.533	0.035
	15–18	12.67^a^	9.61^b^	11.43^a^	10.84^ab^	13.12^a^	5.935	0.026
	Total	13.55^a^	11.17^b^	12.05^ab^	11.47^b^	14.29^a^	8.337	0.051
EM (g/hen)	10–14	319.57 a	208.28 c	256.77 ab	245.92 b	288.78 a	71.82	0.031
	15–18	298.72 a	197.69 c	261.99 ab	245.71 b	276.96 a	53.76	0.023
	Total	618.40 a	440.75 c	533.69 ab	506.16 b	582.94 a	32.98	0.041
HD (%)	10–14	98.90 a	74.53 d	86.11 bc	84.94 c	92.66 b	11.87	0.031
	15–18	91.58 a	72.64 d	86.96 ab	81.63 c	85.71 bc	7.814	0.023
	Total	95.24 a	73.59 c	86.53 bc	83.29 c	89.19 ab	12.65	0.032
HD (egg/hen)	10–14	27.69 a	20.87 c	24.11 ab	23.78 bc	25.95 a	4.873	0.019
	15–18	25.64 a	20.34 b	24.35 a	22.86 ab	24.00 a	9.980	0.029
	Total	53.33 a	44.75 c	49.86 ab	48.00 bc	51.43 a	12.76	0.049
MORT (%)	10–14	2.50 c	13.75 a	8.75 b	7.50 b	7.50 b	4.582	0.015
	15–18	0.00 c	14.49 a	5.48 b	5.41 b	5.41 b	1.172	0.033
	Total	2.50 c	26.25 a	13.75 b	12.50 b	12.50 b	2.021	0.047

CAR, L-carnitine; CrM, creatine monohydrate; G1 -control; G2, 2.5 ppm LA in drinking water (oxidative stress factor); G3, G4, G5, 2.5 ppm LA and adding 500 mg/kg CrM, 500 mg/kg CAR and mixture of 250 mg/ kg CrM + 250 mg/kg Car (CrMCAR) in diet; LA, lead acetate; respectively.

a,b,c,d Means within rows with different letters differ significantly at *P* ≤ 0.05. SEM, standard error mean; BW, body weight; FI, feed intake; WI, water intake; EM, egg mass; FCR, feed conversion ratio; PER, protein efficiency ratio; EER, energy efficiency ratio; HF, hen-day egg production; MORT, mortality.

In Table 3 traits of the whole eggs as well as the eggshell were presented. The egg weight increased with the age of birds in all groups except G2, as well as all groups achieved a higher value of this trait than G2 at 15 to 18 wk (*P* ≤ 0.05). Birds in groups G1 and G4 laid considerably more rounded eggs (EI) than in other groups regardless of the analysis term. Eggs from G1, G4, and G5 were characterized by the highest eggshell weight and thickness compared to G2 and G3. The calculated eggshell parameters (ESA, SV) demonstrated the highest values in G1 and G5 groups, however, the lowest SWUSA was found in G3 concerning higher levels in all other groups.

**Table 3:** The traits of the whole egg and the eggshell of Japanese quails fed with antioxidant factors against the oxidative stress.

Trait	Age (wk)	Group					SEM	*P* value
		G1	G2	G3	G4	G5		
EW (g)	14	11.54 a	9.98 c	10.65 ab	10.34 bc	11.13 a	3.981	0.038
	18	11.65 a	9.72 c	10.76 b	10.75 b	11.54 a	2.953	0.022
	Total	11.59 a	9.85 c	10.71^ab^	10.54 bc	11.33 a	1.843	0.019
EI (%)	14	76.22 a	74.07 b	74.29 b	75.81 a	75.19 ab	27.46	0.035
	18	76.76	75.48	75.15	76.35	75.27	32.98	0.383
	Total	76.79 a	74.89 c	75.01 bc	76.28 a	75.23 b	42.47	0.022
SW (g)	14	1.82 a	1.56 b	1.55 b	1.73 ab	1.84 a	0.053	0.020
	18	1.67 a	1.49 b	1.53 ab	1.65 a	1.63 a	0.030	0.017
	Total	1.75 a	1.53 c	1.54 bc	1.69 ab	1.74 a	0.123	0.032
ST (mm)	14	0.19 a	0.17 b	0.18 ab	0.17 b	0.19 a	0.004	0.032
	18	0.19 a	0.16 b	0.18 ab	0.16 b	0.18 ab	0.008	0.039
	Total	0.19 a	0.17 b	0.18 ab	0.17 b	0.19 a	0.012	0.044
ESA (cm^2^)	14	22.34 a	20.17 b	21.11 a	20.68 ab	21.78 a	10.761	0.018
	18	22.49 a	19.79 b	21.27 a	21.25 ab	22.34 a	10.97	0.037
	Total	22.42 a	19.98 d	21.19 bc	20.97 cd	22.06 ab	5.982	0.043
SV (cm^3^)	14	0.42 a	0.34 b	0.38 a	0.35 ab	0.41 a	0.002	0.038
	18	0.43 a	0.31 c	0.38 ab	0.34 bc	0.40 a	0.004	0.044
	Total	0.43 a	0.33 b	0.39 a	0.35 ab	0.41 a	0.010	0.033
SWUSA	14	3.96 a	3.93 ab	3.69 b	4.18 a	4.15 a	1.130	0.028
	18	3.60	3.85	3.57	3.86	3.56	1.531	0.191
	Total	3.78 ab	3.89 a	3.62 b	4.03 a	3.85 a	1.740	0.025

CAR, L-carnitine; CrM, creatine monohydrate; G1 -control; G2, 2.5 ppm LA in drinking water (oxidative stress factor); G3, G4, G5, 2.5 ppm LA and adding 500 mg/kg CrM, 500 mg/kg CAR and mixture of 250 mg/ kg CrM + 250 mg/kg Car (CrMCAR) in diet; LA, lead acetate; respectively.

a,b,c,d Means within rows with different letters differ significantly at *P* ≤ 0.05. SEM, standard error mean; EW, egg weight; EI, egg index; SW, eggshell weight; ST, eggshell thickness; ESA, egg surface area; SV, eggshell volume; SWUSA, eggshell weight per unit of surface area.

The egg content traits were presented in Table 4. Considerably worse (*P* ≤ 0.05) quality of albumen, measured by its height (AH) and Haugh units, was demonstrated by eggs derived from G2 and G3 groups, stayed under oxidative stress induced and without supplementation or supplemented with CrM. The best quality of this egg element characterized eggs from negative control (G1) and G5. At the same time, significantly the biggest yolks (YW, YI) and more intensively colored yolk (YC) were found in G1, G4, and G5 concerning other groups.

**Table 4:** The traits of the egg content of Japanese quails fed with antioxidant factors against the oxidative stress.

Trait	Age (wk)	Group					SEM	*P* value
		G1	G2	G3	G4	G5		
AW (g)	14	6.19 a	4.99 b	5.69 a	5.52 ab	5.62 a	1.840	0.044
	18	6.33 a	4.89 b	5.76 a	5.11 ab	5.83 a	2.403	0.029
	Total	6.26 a	4.94 b	5.73 a	5.32 ab	5.73 a	1.042	0.051
AH (mm)	14	4.24 a	3.34 c	3.59 bc	3.69 ab	4.02 a	1.112	0.052
	18	4.21 a	3.14 c	3.42 bc	3.44 ab	4.00 a	1.311	0.050
	Total	4.23 a	3.24 c	3.51 bc	3.56 ab	4.01 a	1.698	0.023
HU	14	88.29 a	84.23 c	85.19 bc	86.06 ab	87.35 a	11.09	0.049
	18	88.04 a	83.23 b	84.04 ab	84.05 ab	86.91 a	3.093	0.053
	Total	88.17 a	83.74 c	84.62 bc	85.06 ab	87.13 a	9.650	0.041
YW (g)	14	3.53 ab	3.43 b	3.41 bc	3.09 c	3.67 a	1.095	0.046
	18	3.65 ab	3.34 c	3.47 bc	3.99 a	4.08 a	1.174	0.039
	Total	3.59 a	3.39 b	3.44 ab	3.54 a	3.88 a	1.632	0.048
YI (%)	14	47.41 a	44.09 b	45.76 ab	46.15 a	46.99 a	11.642	0.053
	18	45.83 a	43.56 c	44.53 ab	44.82 a	44.16 bc	10.630	0.048
	Total	46.54 a	43.83 b	45.07 ab	45.41 a	45.57 a	9.805	0.052
YC (pts)	14	11.54 a	9.25 c	9.98 bc	10.34 a	10.13 ab	4.431	0.042
	18	11.65 a	8.97 c	10.16 b	10.15 b	10.20 ab	2.760	0.033
	Total	11.60 a	9.11 b	10.07 ab	10.25 a	10.17 a	1.870	0.038

CrM, creatine monohydrate; CAR, L-carnitine; G1 -control; G2, 2.5 ppm LA in drinking water (oxidative stress factor); G3, G4, G5, 2.5 ppm LA and adding 500 mg/kg CrM, 500 mg/kg CAR and mixture of 250 mg/ kg CrM + 250 mg/kg Car (CrMCAR) in diet; LA, lead acetate; respectively.

a,b,c Means within rows with different letters differ significantly at *P* ≤ 0.05. SEM, standard error mean; AW, albumen weight; AH, albumen height; HU, Haugh units; YW, yolk weight; YI, yolk index; YC, yolk color.

Based on results of blood and serum analysis shown in Table 5 the decrease (*P* ≤ 0.05) in H/L was only present in G5 and G1 compared to G2. In comparison to G2, there was a lowering in LOOH and MDA levels with an increase of FRAP and CAT in serum of the G4, G5, and G1 groups. Also, GSH was enhanced by all experimental groups compared to positive control (G2), which stayed under oxidative stress, whereas elevated values of SOD and GPx were achieved by G1, G3, and G5. The lowering (*P* ≤ 0.05) of MDA in egg albumen was registered in G1 and G5 without any differences in this variable in egg yolk. Decreased level (*P* ≤ 0.05) of TAC in egg albumen was for G2 and G4 and in G2 and G3 in yolk.

**Table 5:** The stress biomarkers in blood and egg of Japanese quails fed with antioxidant factors against the oxidative stress.

Trait	Group					SEM	*P* value
	G1	G2	G3	G4	G5		
Blood/serum							
H/L	0.29 c	0.39 a	0.34 ab	0.36 a	0.30 bc	0.002	0.046
LOOH (μmol/L)	36.14 c	47.59 a	40.74 ab	38.44 b	38.36 bc	18.98	0.051
FRAP (μmol/L)	358.63 a	264.44 c	273.64 c	335.63 b	339.89 ab	27.98	0.048
MDA (μmol/L)	2.42 c	3.93 a	3.82 ab	2.62 bc	2.39 c	1.003	0.031
GSH (μmol/L)	66.32 a	44.51 c	49.59 b	56.42 ab	60.56 a	31.87	0.035
SOD (U/mL)	264.61 a	233.21 c	249.71 ab	245.17 bc	252.67 a	45.28	0.040
CAT (U/mL)	692.15 a	489.34 c	491.43 c	590.26 b	612.42 ab	72.98	0.029
GPx (U/L)	2.11 a	1.62 b	1.92 a	1.81 ab	1.95 a	0.031	0.017
COR (µg/dL)	0.07	0.14	0.08	0.09	0.08	0.005	0.094
Egg albumen							
MDA (nM/mg protein)	1.51 b	2.15 a	1.85 ab	1.93 ab	1.71 b	0.321	0.027
TAC (mg GAE/g)	2.52 a	1.62 c	2.00^a^	1.63 bc	1.99^ab^	0.120	0.041
Egg yolk							
MDA (nM/mg protein)	2.12	2.57	2.23	2.34	2.15	0.776	0.081
TAC (mg GAE/g)	3.21^a^	2.52^c^	2.76^bc^	2.87^b^	2.98^ab^	0.121	0.033

CrM, creatine monohydrate; CAR, L-carnitine; G1 -control; G2, 2.5 ppm LA in drinking water (oxidative stress factor); G3, G4, G5, 2.5 ppm LA and adding 500 mg/kg CrM, 500 mg/kg CAR and mixture of 250 mg/ kg CrM + 250 mg/kg Car (CrMCAR) in diet; LA, lead acetate; respectively.

a,b,c Means within rows with different letters differ significantly at *P* ≤ 0.05. SEM, standard error mean; H/L, heterophil to lymphocyte ratio; LOOH, lipid hydroperoxide; FRAP, ferric-reducing ability of plasma; MDA, malondialdehyde; GSH, glutathione; SOD, superoxide dismutase; CAT, catalase; GPx, glutathione peroxidase; COR, corticosterone; TAC, total antioxidant activity.

Table 6 indicates that the significant increase of TP in serum was in favor of all treated groups (G3, G4, G5) and negative control (G1) compared to positive one. Low values of glucose, uric acid and AST were for G4, G5, and G1. The G3 and G5 obtained a lowering (*P* ≤ 0.05) in CHOL and CPK with high Ca values, their levels were similar to those in G1. Besides, a lower ALT, ALP and GGT activity (*P* ≤ 0.05) was only reported in G5 and G1 (negative control). LA (oxidative stress factor) caused a significant decrease in P level in G2 compared to G1, opposite trends were shown by CREA.

**Table 6:** The biochemical indicators in blood serum of Japanese quails fed with antioxidant factors against the oxidative stress.

Trait	Group					SEM	*P* value
	G1	G2	G3	G4	G5		
TP (g/dL)	5.39 a	4.12 b	5.11 a	5.21 a	5.01 a	2.076	0.038
GLU (mg/dL)	204.23 c	220.38 a	215.11 ab	212.81 b	208.15 bc	43.98	0.045
CHOL (mg/dL)	180.62 c	189.38 a	185.67 b	187.02 ab	182.81 bc	38.65	0.036
Ca (mg/dL)	24.43 a	20.37 c	22.61 a	21.73 bc	22.46 ab	9.651	0.042
P (mg/dL)	5.16 a	4.23 b	4.65 b	4.24 b	4.76 ab	1.654	0.037
CREA (mg/dL)	1.44 b	1.72 a	1.56 ab	1.53 b	1.61 ab	0.121	0.049
UA (mg/dL)	3.99 c	5.49 a	4.62 ab	4.51 b	4.06 bc	0.531	0.036
ALT (U/L)	25.26 c	34.61 a	34.17 a	30.21 ab	28.62 bc	9.982	0.023
AST (U/L)	140.33 c	159.21 a	156.39 a	149.25 b	148.25^b^	51.87	0.035
ALP (U/L)	180.28 b	192.64 a	182.47^ab^	191.52 a	180.38 b	23.54	0.052
CPK (U/L)	138.62 c	150.47 a	140.21 b	147.72 ab	139.42 bc	42.98	0.046
GGT (U/L)	6.25 c	7.69 a	7.24 a	7.17 ab	6.64^bc^	2.982	0.039

CrM, creatine monohydrate; CAR, L-carnitine; G1 -control; G2, 2.5 ppm LA in drinking water (oxidative stress factor); G3, G4, G5, 2.5 ppm LA and adding 500 mg/kg CrM, 500 mg/kg CAR and mixture of 250 mg/ kg CrM + 250 mg/kg Car (CrMCAR) in diet; LA, lead acetate; respectively.

a,b,c Means within rows with different letters differ significantly at *P* ≤ 0.05. SEM, standard error mean; TP, total protein; GLU, glucose; CHOL, cholesterol; Ca, calcium; P, phosphorus; CREA, creatinine; UA, uric acid; ALT, alanine aminotransferase; AST, aspartate transaminase; ALP, alkaline phosphatase; CPK, creatine phosphokinase; GGT, gamma-glutamyl transferase.

As it was shown in Table 7, the higher (*P* ≤ 0.05) average level of serum hormones (E2, FSH, and LH) in the G4 and G5 groups was stated. The progesterone (P4) level was also significantly higher but only in G4. In mentioned groups, these indices had similar values as in G1 in comparison to G2.

**Table 7:** The sex hormones levels in blood serum of Japanese quails fed with antioxidant factors against the oxidative stress.

Trait	Group					SEM	*P* value
	G1	G2	G3	G4	G5		
E2 (pg/mL)	72.35 a	65.37 b	69.27 ab	70.34 a	70.51 a	21.74	0.029
P4 (ng/mL)	1.29 a	0.73 b	0.88 ab	1.07^a^	0.85 ab	0.010	0.042
FSH (pg/mL)	274.35 a	198.53 d	200.64 cd	239.65 bc	243.54 b	55.98	0.039
LH (pg/mL)	1599.63 a	1461.25 c	1482.74 c	1524.75 b	1582.62 ab	32.34	0.024

CrM, creatine monohydrate; CAR, L-carnitine; G1 -control; G2, 2.5 ppm LA in drinking water (oxidative stress factor); G3, G4, G5, 2.5 ppm LA and adding 500 mg/kg CrM, 500 mg/kg CAR and mixture of 250 mg/ kg CrM + 250 mg/kg Car (CrMCAR) in diet; LA, lead acetate; respectively.

a,b,c,d Means within rows with different letters differ significantly at *P* ≤ 0.05. SEM, standard error mean; E2, estradiol; P4, progesterone; FSH, follicle-stimulating hormone; LH, luteinizing hormone.

According to data presented in Table 8, a significantly lower count of *E. coli* in groups in which the oxidative stress was induced (G3–G5) was found as well as in G1 in comparison to G2 positive control. Elevated count (*P* ≤ 0.05) of lactic acid bacteria was presented by G3, G5, and G1 compared to G2. In addition, the lowest counts (*P* ≤ 0.05) of total coliform and total aerobic bacteria were indicated by G5 and G1 than G2. Similar relationships between groups were stated in the case of total fungi. No significant differences were documented among groups regarding *Salmonella* spp. count. The change of pH was only found in gizzard, the decrease (*P* ≤ 0.05) of this parameter was registered for G4 and G1 compared to G2. Moreover, no change among groups was in small and large intestines pH.

**Table 8:** Ileal microflora counts (log10 CFU/g of wet weight) and gut pH of Japanese quails fed with antioxidant factors against the oxidative stress.

Trait		Group					SEM	*P* value
		G1	G2	G3	G4	G5		
*Escherichia coli*		1.98 b	3.02 a	2.18 ab	2.11 ab	2.21 ab	0.930	0.044
Lactic acid bacteria		4.55^a^	3.36^b^	4.27^a^	3.51^ab^	4.49^a^	1.652	0.037
*Salmonella* spp.		3.22	3.53	3.28	3.32	3.28	1.881	0.279
Total coliform		2.59^c^	3.87^a^	3.85^a^	3.03^ab^	2.62^bc^	1.221	0.052
Total aerobic bacteria		8.14 c	10.63 a	8.59 ab	8.47 ab	8.45 bc	2.762	0.025
Total fungi		2.15 b	3.16 a	3.14 a	2.42 ab	2.23 ab	1.110	0.037
pH	Gizzard	5.26 b	5.81 a	5.54 ab	5.30 b	5.35 ab	1.231	0.048
	Small intestine	6.15	6.55	6.42	6.38	6.23	1.340	0.382
	Large intestine	6.43	6.76	6.45	6.77	6.54	2.981	0.199

CrM, creatine monohydrate; CAR, L-carnitine; G1, control; G2, 2.5 ppm LA in drinking water (oxidative stress factor); G3, G4, G5, 2.5 ppm LA and adding 500 mg/kg CrM, 500 mg/kg CAR and mixture of 250 mg/ kg CrM + 250 mg/kg Car (CrMCAR) in diet; LA, lead acetate; respectively.

a,b,c Means within rows with different letters differ significantly at *P* ≤ 0.05. SEM, standard error mean.

## DISCUSSION

The ameliorative effects of CrMCAR followed by CAR and CrM in G5, G4, and G3, respectively, were obvious to counteract the OS incidence by optimizing the overall productive performance in birds during all experimental periods. This might be due to the active interplay between CrM and CAR. It was found that rapidly absorbed creatine (**Cr**) and its chemical forms biologically support the growth by different mechanisms such as stimulating the digestive enzymes and preserving the metabolic activity of necessary energy (Wyss and Kaddurah-Daouk, 2000). Due to the Cr antioxidant action, it could protect the growing muscles from vulnerability and modulates glycolytic pathways in sarcoplasm (Lawler et al., 2002). These phenomena probably reflected the increases in BW, reduced FI and improved FCRs for G5. Moreover, the marked superiority in HDs and EM parameters in groups with antioxidant factors could be reflected by enhanced levels of serum gonadal steroid sex hormones especially in G5 and G4 (Table 7) affecting directly the eggs production. These alterations might result from the myoblast differentiation through myostatin downregulating and myogenin upregulating with upregulated genes levels of growth hormone and insulin-like growth factor-1 in hepatic or pectoral tissues during feeding Cr as it was indicated for broilers (Chen et al., 2011) and Mulard ducks (Ibrahim et al., 2019). The important molecular action of CrM in stressed birds is probably to preserve metabolic energy by reduced rapid glycolysis and enhanced ATP production in muscle which is governed by down regulated genes of adenosine 5′-monophosphate-activated protein kinase α2 and liver kinase B1 in hepatic tissue (Zhang et al., 2017). Therefore, the modulation of the reproductive system and synthetic genes for egg proteins and lipids are probably indirectly influenced by these molecular events in ovarian and oviductal muscles.

The possible reason for bigger feed consumption (FI) in CAR group might be connected to the considerable improvement of villus height in the small intestine and thus increase nutrient absorption (Eskandani et al., 2022). Besides, it was found that dietary CAR had a stimulatory impact on intestinal enzymes secretion which is responsible for nutrient digestibility and reduction of lipogenesis (Ringseis et al., 2018), although, in our date a numerical superiority in FCRs for G4 compared to G2 was found. Also, it is possible that Cr in CrMCAR is characterized by its fast distribution in plasma and growing tissues, participates in protein synthesis and then causes to increase PER (Ibrahim et al., 2019). Due to the stressful condition during laying periods in G3, G4, and G5, there was a motivating effect on birds which fed these antioxidative supplements to consume escalated amounts of water as a defensive way to mitigate the toxic impacts of LA and decrease the high accumulation of this pollutant in renal and hepatic tissues (Al-Shammari and Batkowska, 2021; Al-Shammari, 2023).

Dietary CrMCAR, CrM, and CAR proved their efficacy to lower MORT. This might be depended upon the activation of CrM for the immune system and defensive immune cells which protect body organisms from carcinogenicity, microbial invasion, and overall inflammatory cases (Lawler et al., 2002; Wu, 2020). In addition, it was shown that increased antibody titers against injurious viral diseases, elevated levels of immunoglobulins toward pathogens and alleviated inflammatory reactions are major positive influences of CAR. This is because of immune-modulating properties of CAR which lead to inhibit apoptosis and improve survivability of birds (Adabi et al., 2011; Rehman et al., 2017; Azizi-Chekosari et al., 2021).

Undoubtedly, the number of available reports is very limited to compare with our data regarding influence of CrM on overall performance or physiological aspects of laying poultry, especially under stressful environments. Nevertheless, in a rare study (Halle et al., 2006), was found that adding 0.5, 1, and 2 g Cr/kg of diet for laying hen hybrids for 13 wk increased FI but with no alteration in laying rate and daily EM. A similar result was obtained by Yalçin et al. (2005) who conveyed that the diet containing CAR at 100 mg/kg alone or in a mixture with 1.5 g humate/kg had no effect in FCR, HD per hen, BW in laying quails, however, the best EW was achieved in the group fed with CAR for 16 wk. Also, in accordance, Mousa et al. (2022) observed that feeding thermally stressed laying hens with 100 mg CAR alone or with 250 mg vitamin C per kg of feed from 29 to 40 wk increased the eggs production, EM and EW by improved FCR with no changed FI.

The improvements in measurements of egg quality in G3, G4, and G5 (Table 3) could result from the fact that additives used exerted their presumably antioxidative roles to modulate the ovarian and oviductal function and stimulate efficient absorption of nutrients transported into the egg. The Cr has advantageously been proposed to metabolize energy after its recycling to produce ATP through CPK in muscular tissues. Also, it might protect against oxidative, apoptotic, and toxic reactions in most the vital tissues such as the ovary (Wu, 2020). Most data focused on the supplementation of GAA in the diet as precursor and substrate for Cr endogenous biosynthesis in poultry due to its relatively low cost. For instance, Raei et al. (2020) noticed that various levels of GAA (0.6, 1.2, and 1.8 g/kg) from 56 to 154 d of age incorporated in quails diet potentiated serum and liver antioxidant capacity which reflects on increased EW and YW with no effect on ST, HU, EI and SW depending on used dose. In ISA Brown laying hens, Salah et al. (2020) suggested that was a linear increase in the egg quality involving SW, ST, YW, YI and HU affected by feeding 0.5, 1.0, and 1.5 g of GAA/kg diet for 6 wk due to improved antioxidant and energy metabolism in the liver. Optimal metabolic rate in the magnum by synthesis and secretion of β-ovomucin is responsible for the gelatinous structure of the thick egg albumen could be controlled by CAR received in diet (Ghods-Alavi et al., 2010) and that was visible to optimize HU and AH in current data. Increased YW, YI and YC might be attributed to the stimulatory influence of CAR in the formation of yolk precursors in the hepatic tissue and then activation of their transport to growing follicles and mature oocytes from the synthesis sites in the liver (Ringseis et al., 2018). Additionally, CAR performs its crucial role in the active metabolism of uterine shell glands (Rouhanipour et al., 2022) and stimulates higher absorption of vitamin D3 and circulating Ca that are both indispensable in the integrity of eggshells and reduces evaporation level of CO_2_ from eggshell pores (Ghods-Alavi et al., 2010). Therefore, these changings resulted in an improvement of SW in our data. Similarly, in part, Celik et al. (2004) established that adding 50 mg CAR/L of drinking water played an indispensable role in the albumen quality improvement (AW and AH) with stable ST, although no changes were recorded in EW, YW, SW, YI, EI, YC, and YW in heat-stressed laying hens. In other data, Parizadian et al. (2011) observed that supplementation with CAR (125, 250, and 500 mg/kg) in a diet of quails for 35 to 70 d did not change EW, YW, AH, YI, HU, SW.

The stimulatory effects of supplements applied in G3, G4, and G5 groups on serum redox status through lowering lipid peroxidation indicators (LOOH, MDA), increasing antioxidant enzymes (SOD, CAT, GPx), FRAP and GSH with decreased H/L were obvious. The same effect of antioxidant activity was visible in these groups in the increase of albumen and yolk TAC and decrease albumen MDA in comparison to G2. The impaired antioxidant profile in G2 was probably related to the adverse role of lead by enhanced levels of gene expression for apoptotic proteins such as caspase-3 and caspase-9 proteins in laying quail liver, these proteins induce apoptosis and damage of cellular proteins (Arslan et al., 2022). It was proved by many results that continuous induction of OS by LA (Al-Shammari and Batkowska, 2021) or H_2_O_2_ (Al-Shammari et al., 2019a) as oxidative stress factors has resulted in a weakening of the antioxidant protective system by an increase of lipid peroxidation and inhibition of antioxidant enzymes in poultry blood. Some injurious free radicals including peroxide anions, peroxynitrite and others, that are synthesized in skeletal muscles and brain tissue, could be deactivated or quenched through an antioxidive mechanism attributed to Cr property. These free radicals mostly participate in detrimental events of mitochondrial impairment such as shortage of cellular ATP and dysfunction in buffering capacity of body energy that leads to neurological disorders and congestive heart failure (Wyss and Kaddurah-Daouk, 2000, Wu, 2020). Contrary, Wang et al. (2015) reported that were no alterations in GPx and SOD activity in muscles of 3 h of transport-stressed male broilers during summer which were fed with 600 or 1,200 mg CrM per kg in diet. Our results corresponded to Zhang et al. (2014) who indicated no effect of 600 or 1,200 mg/kg CrM added to the diet on plasma COR in 3 h transport induced stress of male broilers at 42 d of age. The H/L constitutes an active indicator to show stress response in birds, its higher value refers to exposure to stress conditions. The lowering value of H/L in G5 might partially be due to influential CrM in this mixture. Al-Tamimi et al. (2019) found that H/L was inhibited by adding 1.2 g CrM /kg singly or in combination with 1 g betaine/kg of diet at 5 wk of age of acute thermal-stressed chickens. However, in current data, the individual CrM tend to have a numerical decrease only in this value. Improved redox status in G4 might belong to the antioxidative power of CAR. Many authors (Adabi et al., 2011; Surai, 2015; Rehman et al., 2017) suggested that the antioxidant system of CAR in the human or avian bodies is strictly based on various routes including immediate suppression or damaging of free radicals, metals chelating which generates the reactive species, deactivating the special prooxidant enzymes, production of multiple antioxidant enzymes, regulation of heat shock proteins, modulating of vitagenes as well as activation of redox-signaling (Nrf2 and PPARα) which could preserve mitochondrial integrity. In accordance, Çetin and Güçlü (2020) proved that 200 mg/kg CAR alone or with different energy levels, applied in layer hens diet mitigated the OS induced by high stocking density through the increased antioxidant activity of erythrocyte GPx, SOD and CAT with inhibited plasma MDA value compared to control. In Hy-Line layers, it was reported that feeding with 100 mg CAR per kg reduced MDA content with no effect on total SOD and GPx in serum and unchangeable total antioxidant capacity at 50 wk of age (Eskandani et al., 2022). The highest values of TAC content in egg yolk and albumen in G5 give evidence that egg represents a potential pathway for redox reaction by active transport of feed supplements from the diet to main egg components (Surai and Earle-Payne, 2022). Thus, CrMCAR decreased the oxidative susceptibility of the eggs and improved oxidative stability. Correspondingly, Rouhanipour et al. (2022) proved that the total effect of CAR (100, 200 mg/kg) added to a diet enriched with 2 levels of n-3 fatty acids did not alter MDA value in the eggs yolk at 44th wk of Lohmann hens age. This was also in line with Wang et al. (2021), who found that offering dietary 1, 10, and 100 mg Pb nitrate as a stressor per kg BW of ISA Brown layers led to its fast accumulation in eggshell and yolk which may negatively affect antioxidant egg profile and laying rate.

Amelioration in values of biochemical metabolites (TP, GLU, CHOL, Ca, CREA, and UA) and enzymatic values (ALT, AST, ALP, CPK, and GGT) in the serum of G3 to G5 groups might be driven by the upgrading of antioxidative markers in blood serum which investigated during the experiment. It could be suggested that CrM, CAR and their mixture played key roles by maintaining TP content with hyperglycemia influence in serum through their possible mechanism as antioxidants in the blood. Synergistically, CAR in CrMCAR might also inhibit the expression of the 3-hydroxy-3-methylglutaryl coenzyme A reductase activity with increased expression of cholesterol 7 alpha-hydroxylase which are considered as regulatory enzymes for cholesterol metabolism in liver and serum (Rehman et al., 2017; Eskandani et al., 2022). The decrease in measured enzymes especially in G5 might belong to CrM influencing the protection from free radicals production and providing suitable energy demands during oxidative muscle metabolism (Wyss and Kaddurah-Daouk, 2000). Thus, it is clear that the overproduction of hepatic enzymes in the blood occurs during damage in hepatocytes and hepatotoxicity (Estévez, 2015) induced by increasing the electron leakage from mitochondria which generate free radicals. Also, enhanced serum TP and reduced CHOL in G3 could be related to fat-burning properties and accelerated nitrogen metabolism of CrM. Consistent results proved that there was no effect of 3% Cr offered to the broiler diet at 42 d of age in changing serum CREA and ALT content in the liver (Chen et al., 2011). It was shown that CAR maximized liver and kidney functions in poultry by mitochondrial protection from inflammation associated with harmful products (Arslan, 2006; Adabi et al., 2011). This could explain the lower value of CREA and UA in G4. Identical data, in stressed poultry, reported increased values of blood ALT, CREA and UA due to an inappropriate antioxidant defense network (Al-Shammari et al., 2019b; Arslan et al., 2022). Decreased Ca level for G2 was in conjunction with low eggshell quality (Table 3) and this could typically attribute to the influence of LA on osteoporosis function via osteoclast activation and osteoblast deactivation which makes layer hen bones more prone to fractures risk (Wang et al., 2021). Our data were in line with Yalçin et al. (2005) who suggested that was no effect of dietary CAR in serum ALT, ALP and CPK and CHOL in laying quails. Differently, Parizadian et al. (2011) indicated low CHOL and unaltered glucose in the serum of laying quail fed with CAR supplementation.

Increased levels of reproductive hormones in stressed groups (G4, G5) are probably linked to activation of the hypothalamic-pituitary-ovarian axis (**HPO**) in response to feeding by CrMCAR and CAR. The metabolic activity of ovary and oviduct function is modulated by a molecular mechanism of steroid (E2 and P4) and pituitary gland (FSH and LH) hormones in laying hens (Zhao et al., 2023). Thus, the enhancement in values of these sex hormones is probably correlated with promoting more intensive egg production and their high quality in our study. It is reasonable to postulate that dietary CAR, individually or in combination with other feed supplements, protects oocytes membranes from injurious attacks of free radicals and provides energy supply to growing oocytes and boosting the sex hormone secretions through HPO (Agarwal et al., 2018). Differently as indicated by Kazemi-Fard et al. (2015), the level of plasma E2 was unaffected in Hy-Line W-36 layers by feeding 50, 100, and 150 mg CAR/kg of diet for 6 wk except for plasma P4 level which reduced its biosynthesis by 100 mg CAR/kg of diet.

The improved growth of lactic acid bacteria and impaired growth of total coliform as well as total aerobic bacteria in the ileum section of G5 was proved in Table 8, however, no effect in gut pH was induced by G5. This was probably related to the positive effect of this mixture on the eubiosis of gut microbiota and thus enhanced PER and EER which give an indicator for the powerful apparent digestibility of digestible nutrients (Al-Shammari, 2023). It is known that metabolic aspects, secretion of digestive juices, resistance to multiple stressors and toxic substances and expulsion of harmful pathogens are strictly correlated with beneficial microflora population in the gut of avian species (Shang et al., 2018). Besides, the dangerous reactive species are naturally suppressed by antioxidant and anti-inflammatory defense mechanism of commensal gut microbiota inhabited in epithelial mucosa through increasing many enzymatic and nonenzymatic antioxidants. Thus, homeostasis and complexity of microbiota affects gut integrity and health potentially in poultry (Wickramasuriya et al., 2022). Azizi-Chekosari et al. (2021) confirmed that a level of 400 CAR mg/kg of diet improved the numeration of lactobacilli and inhibited *E. coli* and coliforms generally in broilers cecum at 42 d of birds’ age which is inconsistent with our results.

## CONCLUSIONS

It was worth to notice that synergistic level of CrMCAR (250 mg of CrM + 250 mg of CAR per kg of diet) followed by using 500 mg of CAR or CrM per kg of diet separately had an alleviative effect on serum and egg antioxidant properties of laying quails exposed to oxidative stress induced by 2.5 ppm LA in drinking water. Furthermore, the biochemical metabolites as well as the liver enzymatic activity, sex hormones levels and ileal microflora were improved by these feed additives. In general, these collectively pivotal changes led to final improvement in productive performance and egg quality during 8 wk of application.

## DISCLOSURES

The authors declare that they have no known competing financial interests or personal relationships that could have appeared to influence the work reported in the present study.

## References

[bib0001] Adabi S.G., Cooper R.G., Ceylan N., Corduk M. (2011). L-carnitine and its functional effects in poultry nutrition. Worlds Poult. Sci. J..

[bib0002] Aebi H. (1984). Catalase *in vitro*. Meth. Enzymol..

[bib0003] Agarwal A., Sengupta P., Durairajanayagam D. (2018). Role of L-carnitine in female infertility. Reprod. Biol. Endocrinol..

[bib0007] Al-Shammari K.I.A. (2023). Alleviating the oxidative stress in Japanese quails fed L- carnitine and creatine monohydrate through impacts on productive performance, ileal microflora, digestibility and redox system. Vet. Integr. Sci..

[bib0006] Al-Shammari K.I.A., Batkowska J. (2021). The antioxidative impact of dietary vinegar and rocket salad on the productivity, serum oxidation system, and duodenal histology of chickens. Animals.

[bib0004] Al-Shammari K.I.A., Batkowska J., Zamil S.J. (2019). Role of pomegranate peels and black pepper powder and their mixture in alleviating the oxidative stress in broiler chickens. Int. J. Poult. Sci..

[bib0005] Al-Shammari K.I.A., Zamil S .J., Mohammed E.M. (2019). Influence of dietary epigallocatechin-3 gallate and L-arginine and its combination on early laying performance and status of stressed Japanese quails. J. Phys. Conf. Ser..

[bib0008] Al-Tamimi H., Mahmoud K., Al-Dawood A., Nusairat B., Khalaf H.B. (2019). Thermotolerance of broiler chicks ingesting dietary betaine and/or creatine. Animals.

[bib0009] Arslan C. (2006). L-carnitine and its use as a feed additive in poultry feeding a review. Rev. Med. Vet..

[bib0010] Arslan A.S., Seven I., Mutlu S.I., Arkali G., Birben N., Seven P.T. (2022). Potential ameliorative effect of dietary quercetin against lead-induced oxidative stress, biochemical changes, and apoptosis in laying Japanese quails. Ecotoxicol. Environ. Saf..

[bib0011] Azizi-Chekosari M., Bouyeh M., Seidavi A., Ventura M.R. (2021). Effect of dietary supplementation with L-carnitine and fenofibrate on broiler chickens. S. Afr. J. Anim. Sci..

[bib0012] Benzie I.F., Strain J.J (1996). The ferric reducing ability of plasma (FRAP) as a measure of "antioxidant power": the FRAP assay. Anal. Biochem..

[bib0013] Bryant M.P., Burkey L.A. (1953). Cultural methods and some characteristics of some of the more numerous groups of bacteria in the bovine rumen. J. Dairy Sci..

[bib0014] Burtis C.A., Ashwood E.R. (1999).

[bib0015] Burton R., Guion C.W. (1968). The differential leucocyte blood count: its precision and individuality in the chicken. Poult. Sci..

[bib0016] Celik L.B., Tekeli A., Ozturkcan O. (2004). Effects of supplemental L-carnitine in drinking water on performance and egg quality of laying hens exposed to a high ambient temperature. J. Anim. Physiol. Anim. Nutr..

[bib0017] Çetin E., Güçlü B.K. (2020). Effect of dietary L-carnitine supplementation and energy level on oxidant/antioxidant balance in laying hens subjected to high stocking density. J. Anim. Physiol. Anim. Nutr..

[bib0018] Chaveerach P., Keuzenkamp D.A., Lipman L.J., Van K.F. (2004). Effect of organic acids in drinking water for young broilers on Campylobacter infection, volatile fatty acid pro-duction, gut microflora and histological cell changes. Poult. Sci..

[bib0019] Chen J., Wang M., Kong Y., Ma H., Zou S. (2011). Comparison of the novel compounds creatine and pyruvate on lipid and protein metabolism in broiler chickens. Animal.

[bib0020] Draper H.H., Hadeley M. (1990). Malondialdehyde determination as index of lipid peroxidation. Meth. Enzymol..

[bib0021] Duman M., Şekeroğlu A., Yıldırım A., Eleroğlu H., Camcı Ö. (2016). Relation between egg shape index and egg quality characteristics. Eur. Poult. Sci..

[bib0022] Duncan D.B. (1955). Multiple range test and multiple F test. Biometrics.

[bib0023] Eisen E.J., Bohren B.B., McKean H.E. (1962). The Haugh unit as a measure of egg albumen quality. Poult. Sci..

[bib0024] Eskandani M., Navidshad B., Eskandani M., Vandghanooni S., Aghjehgheshlagh F.M., Nobakht A., Shahbazfar A.A. (2022). The effects of L-carnitine-loaded solid lipid nanoparticles on performance, antioxidant parameters, and expression of genes associated with cholesterol metabolism in laying hens. Poult. Sci..

[bib0025] Estévez M. (2015). Oxidative damage to poultry: from farm to fork. Poult. Sci..

[bib0026] Ghods-Alavi B., Samie H., Jahanian R. (2010). Effects of supplementary dietary L-carnitine on performance and egg quality of laying hens fed diets different in fat level. Ital. J. Anim. Sci..

[bib0027] Halle I., Henningm M., Kohler P. (2006). Studies of the effects of creatine on performance of laying hens, on growth and carcass quality of broilers. Landbauforschung-Ger.

[bib0028] Ibrahim D., El Sayeda R., Abdelfattah-Hassan A., Morshedyd A.M. (2019). Creatine or guanidinoacetic acid? Which is more effective at enhancing growth, tissue creatine stores, quality of meat, and genes controlling growth/myogenesis in Mulard ducks. J. Appl. Anim. Res..

[bib0029] Kazemi-Fard M., Yousefi S., Dirandeh E., Rezaei M. (2015). Effect of different levels of L-carnitine on the productive performance, egg quality, blood parameters and egg yolk cholesterol in laying hens. Poult. Sci. J..

[bib0030] Lawler J.M., Barnes W.S., Wu G., Song W., Demaree S. (2002). Direct antioxidant properties of creatine. Biochem. Biophys. Res. Commun..

[bib0031] Manafi M., Khalaji S., Hedayati M. (2016). Assessment of a probiotic containing Bacillus subtilis on the performance and gut health of laying Japanese quails (*Coturnix Coturnix Japonica*). Braz. J. Poult. Sci..

[bib0032] Misra H.P., Fridovich I. (1972). The role of superoxide anion in the autooxidation of epinephrine and a simple assay for superoxide dismutase. Biol. Chem..

[bib0033] Mousa M.A.M., Khosht A.R., Eshera A.A.M. (2022). Effect of chromium chloride or L-carnitine supplementation either alone or combination with vitamin C on productive performance of golden Montazah chickens during the summer season. Egypt. Poult. Sci..

[bib0034] Muchacka R., Sosnówka-Czajka E., Skomorucha I., Kapusta E., Greń A. (2018). The activity of antioxidant enzymes in blood plasma and eggs of laying hens kept in various rearing systems during the summer heat period. Eur. Poult. Sci..

[bib0035] NRC. National Research Council (1994).

[bib0036] Parizadian B., Ahangari Y.J., Shargh M.S., Sardarzadeh A. (2011). Effects of different levels of L-carnitine supplementation on egg quality and blood parameters of laying Japanese quail. Int. J. Poult. Sci..

[bib0037] Raei A., Karimi A., Sadeghi A. (2020). Performance, antioxidant status, nutrient retention and serum profile responses of laying Japanese quails to increasing addition levels of dietary guanidinoacetic acid. Ital. J. Anim. Sci..

[bib0038] Rehman Z., Naz S., Khan R.U., Tahir M. (2017). An update on potential applications of L-carnitine in poultry. Worlds Poult. Sci. J..

[bib0039] Reitman S., Frankel S. (1957). A colorimetric method for the determination of serum glutamic oxalacetic and glutamic pyruvic transaminases. Am. J. Clin. Pathol..

[bib0040] Ringseis R., Keller J., Eder K. (2018). Basic mechanisms of the regulation of L-carnitine status in monogastrics and efficacy of L-carnitine as a feed additive in pigs and poultry. J. Anim. Physiol. Anim. Nutr..

[bib0041] Rouhanipour H., Sharifi S.D., Irajian G., Jalal M.P. (2022). The effect of adding L-carnitine to omega-3 fatty acid diets on productive performance, oxidative stability, cholesterol content, and yolk fatty acid profiles in laying hens. Poult. Sci..

[bib0042] Salah A.S., Ahmed-Farid O.A., El-Tarabany M.S. (2020). Effects of guanidinoacetic acid supplements on laying performance, egg quality, liver nitric oxide and energy metabolism in laying hens at the late stage of production. J. Agric. Sci..

[bib0043] Salih A.M., Smith D.M., Price J.F., Dawson L.E. (1987). Modified extraction 2-thiobarbituric acid method for measuring lipid oxidation in poultry. Poult. Sci..

[bib0044] SAS. Statistical Analysis System (2012). User's Guide. Statistical Analysis System. Ver. 9.1.

[bib0045] Shafey T.M. (2002). Effects of egg size and eggshell conductance on hatchability traits of meat and layer breeders flocks. Asian-Australas. J. Anim. Sci..

[bib0046] Shang Y., Kumar S., Oakley B., Kim W.K. (2018). Chicken gut microbiota: importance and detection technology. Front. Vet. Sci..

[bib0047] Södergren E., Nourooz-Zadeh J., Berglund L., Vessby B. (1998). Re-evaluation of the ferrous oxidation in xylenol orange assay for the measurement of plasma lipid hydroperoxides. J. Biochem. Biophys. Methods..

[bib0048] Speak M.L. (1984).

[bib0049] Stanquevis C.E., Furlan A.C., Marcato S.M., Oliveira-Bruxel T.M., Perine T.P., Finco E.M., Grecco E.T., Benites M.I., Zancanela V.T. (2021). Calcium and available phosphorus requirements of Japanese quails in early egg-laying stage. Poult. Sci..

[bib0050] Surai P.F. (2015). Antioxidant action of carnitine: molecular mechanisms and practical applications. EC Vet. Sci..

[bib0051] Surai P.F., Earle-Payne K. (2022). Antioxidant defences and redox homeostasis in animals. Antioxidants.

[bib0052] Surai P.F., Kochish I.I., Fisinin V.I., Kidd M.T. (2019). Antioxidant defence systems and oxidative stress in poultry biology: an update. Antioxidants.

[bib0053] Tietz N.W. (1986).

[bib0054] Wang S., Li Q., Gao Y., Zhou Z., Liy Z. (2021). Influences of lead exposure on its accumulation in organs, meat, eggs and bone during laying period of hens. Poult. Sci..

[bib0055] Wang X.F., Zhu X.D., Li Y.J., Liu Y., Li J.L., Gao F., Zhou G.H., Zhang L. (2015). Effect of dietary creatine monohydrate supplementation on muscle lipid peroxidation and antioxidant capacity of transported broilers in summer. Poult. Sci..

[bib0056] Wickramasuriya S.S., Park I., Lee K., Lee Y., Kim W.H., Nam H., Lillehoj H.S. (2022). Role of physiology, immunity, microbiota, and infectious diseases in the gut health of poultry. Vaccines.

[bib0057] Wu G. (2020). Important roles of dietary taurine, creatine, carnosine, anserine and 4-hydroxyproline in human nutrition and health. Amino Acids.

[bib0058] Wyss M., Kaddurah-Daouk R. (2000). Creatine and creatinine metabolism. Physiol. Rev..

[bib0059] Yalçin S., Ergün A., Erol H., Yalçin S., Özsoy B. (2005). Use of L-carnitine and humate in laying quail diets. Acta Vet. Hung..

[bib0060] Young D.S. (2000).

[bib0061] Zhang L., Li J.L., Gao T., Lin M., Wang X.F., Zhu X.D., Gao F., Zhou G.H. (2014). Effects of dietary supplementation with creatine monohydrate during the finishing period on growth performance, carcass traits, meat quality and muscle glycolytic potential of broilers subjected to transport stress. Animal.

[bib0062] Zhang L., Wang X., Li J., Zhu X., Gao F., Zhou G. (2017). Creatine monohydrate enhances energy status and reduces glycolysis via inhibition of AMPK pathway in pectoralis major muscle of transport-stressed broilers. J. Agric. Food Chem..

[bib0063] Zhao J., Pan H., Liu Y., He Y., Shi H., Ge C. (2023). Interacting networks of the hypothalamic–pituitary–ovarian axis regulate layer hens performance. Genes.

